# Pairwise interaction of in-line spheroids settling in a linearly stratified fluid

**DOI:** 10.1007/s00707-024-04125-4

**Published:** 2024-11-04

**Authors:** Abdullah M. Abdal, Lyes Kahouadji, Seungwon Shin, Jalel Chergui, Damir Juric, Colm-Cille P. Caulfield, Omar K. Matar

**Affiliations:** 1 Department of Chemical Engineering, Imperial College London, London, SW7 2AZ UK; 2 Department of Environmental and Sustainability Engineering, College of Engineering and Energy, Abdullah Al Salem University, 12037 Kuwait City, Kuwait; 3 Department of Mechanical and System Design Engineering, Hongik University, Seoul, 04066 Korea; 4 Centre National de la Recherche Scientifique (CNRS), Laboratoire Interdisciplinaire des Sciences du Numérique (LISN), Université Paris Saclay, 91400 Orsay, France; 5 Department of Applied Mathematics and Theoretical Physics, Centre for Mathematical Sciences, University of Cambridge, Wilberforce Road, Cambridge, CB3 0WA UK; 6 Institute for Energy and Environmental Flows, University of Cambridge, Madingley Rise, CB3 0EZ Cambridge, UK

## Abstract

This study investigates the transport of particles in density-stratified fluids, a prevalent natural phenomenon. In the ocean, particles and marine snow descend through fluids with significant density variations due to salinity and temperature gradients. Such heterogeneity in the background fluid affects the settling or rising rates of particles, often leading to accumulation at transitional density layers. Previous research has primarily focused on spherical particles, examining their isolated motion, pairwise interactions, and collective transport in stratified fluids. This work, however, extends the investigation to the interaction between two spheroidal particles settling in-line in a linearly stratified fluid. This study employs an immersed-boundary technique to perform particle-resolved numerical simulations in a three-dimensional Cartesian domain. The results showcase the effects of varying the stratification strength through the Froude number, the particles’ aspect ratios, and the initial separation distance between the particles on the interaction dynamics between the settling spheroids.

## Introduction

Research on the movement of particles, bubbles, and droplets in fluid environments is extensively prevalent, given its widespread occurrence in both natural settings and industrial applications. In the realm of oceanography, the settling of particles and marine snow in fluids, which exhibit substantial density gradients due to variances in salinity and temperature, is a critical phenomenon [46]. This variation in the background fluid’s density has a notable impact on the settling or ascent rates of these entities, potentially causing them to stabilise at layers where density shifts occur.

The study of particulate transport, including solid particles, bubbles, and droplets in homogeneous fluid media, has played a crucial role in multiphase flow research due to its wide range of applications. When considering deformable bodies, their motion can also be characterised based on surface mobility. Surfaces that are immobile, often due to the presence of contaminants like surfactants, achieve smaller translational velocities compared to mobile surfaces [21]. In the viscous regime, the motion of these bodies is characterised by the Stokes based Reynolds number, where $Re_s=\rho _f V_s D_p/\mu _f$. The particle terminal velocity can be estimated by the Stokes equation, such that $V_s=\frac{2}{9}(\rho _p-\rho _f) gR^2/\mu _f$ where $\rho _p$ is the particle density, $R=D_p/2$ is the particle radius, *g* is the gravitational acceleration, and $\rho _f$ and $\mu _f$ are the fluid density and viscosity, respectively. In the inertial regime, hence larger Reynolds numbers $(Re=\rho _f V_f D_p/\mu _f)$, [37] and ten Cate et al. [57] considered the settling dynamics for solid spheres, where $Re \sim \mathcal {O}(10-10^3)$, such that the particle terminal velocity $V_f$ is an emergent property of the flow dynamics.

In engineering contexts, many processes involve an interface between two distinct fluids, influencing the movement of both rigid and deformable bodies. Notable processes in this domain, such as liquid-liquid extraction, encapsulation, and coating procedures, have been studied by Rydberg et al. [48], Hashimoto et al. [30], and Pitois et al. [45]. When considering the study of a two-layer immiscible fluid configuration on the settling dynamics of these bodies, [43, 44] have investigated the transport of spherical particles and [7] have studied the bubble dynamics in three-phase system. For spherical particles in the inertial regime, the settling dynamics are additionally governed by the viscosity ratio between the two fluids, and the surface tension $\sigma ,$ where the particle is seen to drag a column of fluid as it penetrates the initially unperturbed interface. This column of fluid was found to be either axisymmetric or asymmetric, depending on the approach velocity of the particle, and may lead to pinch-off or fragmentation. When considering the rising motion of a bubble through a liquid-liquid interface, the dynamics may lead to changes in the bubble shape which affects the volume of entrained fluid in its wake.

In the opposing limit, a liquid film may form between the interface and the particle, such that the passage of the particle may only be achieved after film drainage is complete [43]. In this regime, the interaction between the particle and the interface is influenced by several factors, including the particle’s approach velocity and its density relative to the surrounding fluid phases. Both experimental and theoretical [28, 29, 53] studies have explored this issue, taking into account the effects of gravity on the thinning of the liquid film between the particle and the interface. When deformable surfaces like drops or bubbles are involved, the situation becomes more complex. In drop-drop or bubble-bubble interactions, deformation of the surfaces can cause asymmetric thinning of the liquid film, which can lead to non-uniform drainage [10].

The settling motion of spherical particles [20], spherical particle pairs [15, 17], spherical particle clusters [9, 14, 19], droplets [3, 4, 13, 50], and bubbles [23] in a density-stratified ambient fluid were previously studied. Stratification of the ambient fluid was found to significantly affect the settling velocity and interaction dynamics of these objects, and the motion of particle clusters or bubbles may lead to sufficient mixing, thereby mitigating stratification effects [6, 31].

[2] have studied the motion of isolated and a pair of spheroidal particles in a homogeneous fluid. At sufficiently large inertia, the isolated particles were found to settle in an oscillatory regime, leading to the formation of different vortices in the spheroid’s wake. For a pair of spheroidal particles, the Drafting-Kissing-Tumbling regime was studied for an oblate and prolate particle pair with varying initial particle orientations.

In a stratified ambient fluid, there were recent studies on the transport of isolated spheroidal particles and discs in either a linear [36, 39] or sharp density gradient [40, 41]. As the particles settle, stratification induced reorientation of the particles was found, such that the equilibrium orientation of the particles shifted from broad-side on to edgewise when compared to the homogeneous fluid counterpart. Most notably, as a particle settles in a stratified fluid, the particle encounters an enhanced drag due to the variation in fluid density, and may not reach a statistically steady state as the particle velocity may oscillate depending on the stratification strength [20].

The dynamics of motion within stratified fluids have been thoroughly reviewed in works by Govindarajan et al. [24], Ardekani et al. [1], Magnaudet et al. [35], and More et al. [38]. Most studies found in the literature have focused on the transport and interaction between spherical particles. However, particles found in nature and industry are not perfectly spherical and may have an irregular shape. For instance, microplastics are generally defined as small, irregularly shaped plastic particles less than 5 mm in size. These particles often manifest as elongated fibres, flat planar fragments [12], or nearly spherical pellets [47]. For modelling purposes, these diverse shapes can be approximated as spheroidal particles [16]. The motivation behind this work is to gain insight into the particle settling dynamics as typically found in the ocean. When considering particle transport outside the Stokes regime, an alternative way of characterising the particle motion in a homogeneous fluid is through the Galilei number (to be defined in Sect. 2), which corresponds to a particle Reynolds number based on a gravitational velocity scale and the diameter of a sphere with the same volume as that of a spheroid. Although some work has been carried out considering the settling of individual spheroidal particles and their pairwise interaction in homogeneous fluids [2], and the settling of these particles in a density-stratified fluid [36, 39], the pairwise interaction between spheroidal particles settling in a density-stratified fluid remains unstudied [56].

This work considers the settling of a pair of initially in-line spheroidal particles in a linearly stratified fluid, investigating the effects of the stratification strength, the particle aspect ratio, and the initial separation distance on the particle settling dynamics. The rest of this paper is organised as follows: Section 2 covers the problem formulation, numerical method, and the validation of the numerical method. Section 3 contains a discussion of the numerical results. Finally, concluding remarks are provided in Sect. 4.

## Methodology

This section contains the problem formulation, governing equations, and the numerical method used to carry out the computations. The particle’s motion and interaction with the fluid are modelled using a fictitious domain method, such that the equations of mass and momentum conservation are expressed using a single-field formulation:

1 $$\begin{aligned}&\displaystyle \nabla \cdot {\textbf {u}}=0, \end{aligned}$$

2 $$\begin{aligned}&\displaystyle \rho \left( \frac{\partial {\textbf {u}}}{\partial t}+{\textbf {u}}\cdot \nabla {\textbf {u}}\right) =-\nabla {p}+\rho {\textbf {g}}+\nabla \cdot \mu \left( \nabla {\textbf {u}} +\nabla {\textbf {u}}^T\right) {+{\textbf {F}}_{FSI}}, \end{aligned}$$

3 $$\begin{aligned}&\displaystyle \frac{\partial \rho _f}{\partial t}+{\textbf {u}}\cdot \nabla \rho _f = \nabla \cdot (\kappa \nabla \rho _f ), \end{aligned}$$

where ${\textbf {u}}$, $\rho$, *p*, $\mu$, ${\textbf {g}}$, ${\textbf {F}}_{FSI}$, $\rho _f$, and $\kappa$ denote the velocity, density, pressure, viscosity, gravitational acceleration, direct forcing term for fluid–structure interaction, unperturbed background fluid density, and diffusivity of the stratifying agent, respectively. In this approach, we assume that with minimal diffusion of the stratifying agent, the velocity field may be considered divergence-free. [20] have demonstrated that in the limit of weak diffusion of the stratifying agent, the local variables, such as the particle velocity, are virtually indistinguishable from those of a Boussinesq fluid.

Within this formulation, the density and viscosity read

4 $$\begin{aligned} \begin{aligned} \rho ({\textbf {x}},t)= \rho _f+(\rho _p-\rho _f)\mathcal {H}({\textbf {x}},t), \end{aligned} \end{aligned}$$

5 $$\begin{aligned} \begin{aligned} \mu ({\textbf {x}},t)=\mu _f+(\mu _p-\mu _f)\mathcal {H}({\textbf {x}},t), \end{aligned} \end{aligned}$$

where the subscripts (*f*, *p*) denote the background liquid, and the solid phase, respectively. A smoothed three-dimensional Heaviside function $\mathcal {H}({\textbf {x}},t)$ is defined as zero in the fluid phase, and unity in the particle. The sharp variation between the two phases is resolved numerically with a steep, yet smooth transition across three to four grid cells [32, 51]. In this work, we assume that the unperturbed background fluid density profile varies linearly with depth when considering the particle pairwise interaction, such that:

6 $$\begin{aligned} \rho _f(z)=\rho _0+\gamma z, \end{aligned}$$

where $\gamma =d\rho _f/dz~<0$ is the constant stratification gradient, $\rho _0$ represents the density at the initial position of the trailing particle, and *z* is the vertical coordinate. We have kept the fluid viscosity $\mu _f$ constant.

Tracking the solid body motion is achieved by introducing a signed distance function $\phi _s$ to the nearest interface. At the start of the simulation, $\phi ^0_{s}$ is calculated at the cell centre of the Eulerian grid $(x^0_{i},y^0_{i},z^0_{i})$ to create the solid area, which is then updated to calculate $\phi ^n_{s}$ at $(x^n_{i},y^n_{i},z^n_{i})$ by tracking the solid’s centre position and rotation over time. The solid object’s movement is updated by its momentum-averaged translational and rotational velocities, which are calculated as follows

7 $$\begin{aligned} m_p {\textbf {u}}_p = \int _{\mathcal {V}} \rho _p {\textbf {u}} d\mathcal {V}, \end{aligned}$$

8 $$\begin{aligned} {\textbf {I}}_p {\varvec{\omega }_p}=\int _{\mathcal {V}} \textbf{r}\times \rho _p {\textbf {u}} d\mathcal {V}, \end{aligned}$$

where $m_p$ is the mass of the solid, ${\textbf {u}}_p$ is its centroid translational velocity, $\mathcal {V}$ is the solid volume, ${\textbf {r}}$ is the radial vector from the particle center, ${\textbf {I}}_p$ is the moment of inertia, and $\varvec{\omega _p}$ is the angular velocity of its centroid. The moment of inertia can be calculated as follows,

9 $$\begin{aligned} {\textbf {I}}_p =\int _{\mathcal {V}} \rho _p |({\textbf {r}}\cdot {\textbf {r}}){\textbf {I}}-{\textbf {r}} \otimes {\textbf {r}}| d\mathcal {V}, \end{aligned}$$

and the velocity field of the solid particle corresponds to

10 $$\begin{aligned} {\textbf {u}}_s (x,y,z)={\textbf {u}}_p + \varvec{\omega }_p \times {\textbf {r}}. \end{aligned}$$

Utilising equation (10), the updated solid distance function can be calculated at the current position $(x^n_{i},y^n_{i},z^n_{i})$ by tracing back to the original position $(x^0_{i},y^0_{i},z^0_{i})$ as follows

11 $$\begin{aligned} \phi _s(x^n,y^n,z^n)=\phi _s(x^n-{u}_s\Delta t,y^n-{v}_s\Delta t,z^n-{w}_s\Delta t)=\phi _s(x^0,y^0,z^0). \end{aligned}$$

In Eq. 11, ${\textbf {u}}_s$ is defined by Eq. 10 such that any individual points *x*, *y*, and *z* of the particle’s current location can be traced back to their initial starting positions. The initial particle shape can be described analytically and thus we can calculate a very accurate distance function field at the start of the simulation. As the simulation progresses, we track the location of the centroid and rotational angle in the *x*, *y*, and *z* directions. Thus at any given time step, we can trace back to the initial point using the current centroid position and angular rotation. The current distance function field is then computed using its initial analytic form.

Rigid body constraints are applied to the solid area for the momentum-averaged translational and rotational velocities, and an artificial slip condition is applied at the solid surface such that an additional high viscosity coefficient is utilised for the solid area. The boundary conditions applied on the solid surface correspond to no-slip and no-flux, which are widely utilised in previous studies of flow over solids in stratified fluids [20, 25, 26, 39, 58]. For the velocity field inside the solid, we force it’s value to be the solid velocity defined by Eq. 10 during the entire simulation. Thus, a non-slip boundary condition is explicitly enforced for the solid particle. Since we then use the one-fluid model for solving the flow in the entire domain for all phases, the velocity field from the one-fluid model flow solution (Eqs. 14–18) might not satisfy the non-slip condition. Therefore we use a relatively high viscosity inside the solid which essentially enforces the non-slip condition. We find that using a viscosity of about 100x that of the liquid is sufficient, and in this work, we utilise a solid viscosity of 500x the viscosity of the fluid.

We scale space, velocity, time, and vorticity on $D_p$, $V_t=\sqrt{D_p|{\rho _p}/{\rho _0}-1|g}$, $\sqrt{D_p/g}$, and $\sqrt{g/D_p}$, respectively, so that the non-dimensional parameters governing the problem are the Galilei, Froude, and Schmidt numbers, respectively given by

12 $$\begin{aligned} Ga=\frac{D_p V_t\rho _0}{\mu _f}, ~~~ Fr=\frac{V_t}{ND_p}, ~~~ Sc=\frac{\mu _f}{\rho _0 \kappa }; \end{aligned}$$

here, $N=\sqrt{{-g \gamma }/{\rho _0}}$ is the Brunt-Väisälä frequency, and the limit $Fr \rightarrow \infty$ corresponds to the unstratified, homogeneous fluid case. In this work, we set $\rho _p=1141$ kg/$\hbox {m}^{3}$ and $\rho _0=1000$ kg/$\hbox {m}^{3}$, and fix the fluid viscosity at $\mu _f=0.001$ kg/ms. We also set $Sc=700$, which corresponds to a salinity-stratified fluid, and obtain solutions for $Ga=4.65$ and $2 \le Fr \le 12$. [34] observed that particles and marine snow in the ocean settle with Reynolds numbers ranging from 0.2 to 23 and Froude numbers between 5 and 70. In this work, we investigate the effects of varying the particle shape on the settling behaviour, considering a particle size and background fluid stratification strengths similar to those found in the ocean. Using the above scaling, the dimensionless version of Eq. (6) reads:

13 $$\begin{aligned} \tilde{\rho _f}=1-\frac{|{\rho _p}/{\rho _0}-1|}{Fr^2}{\tilde{z}}, \end{aligned}$$

where the tildes, which designate dimensionless variables, are suppressed henceforth.

**Fig. 1:**
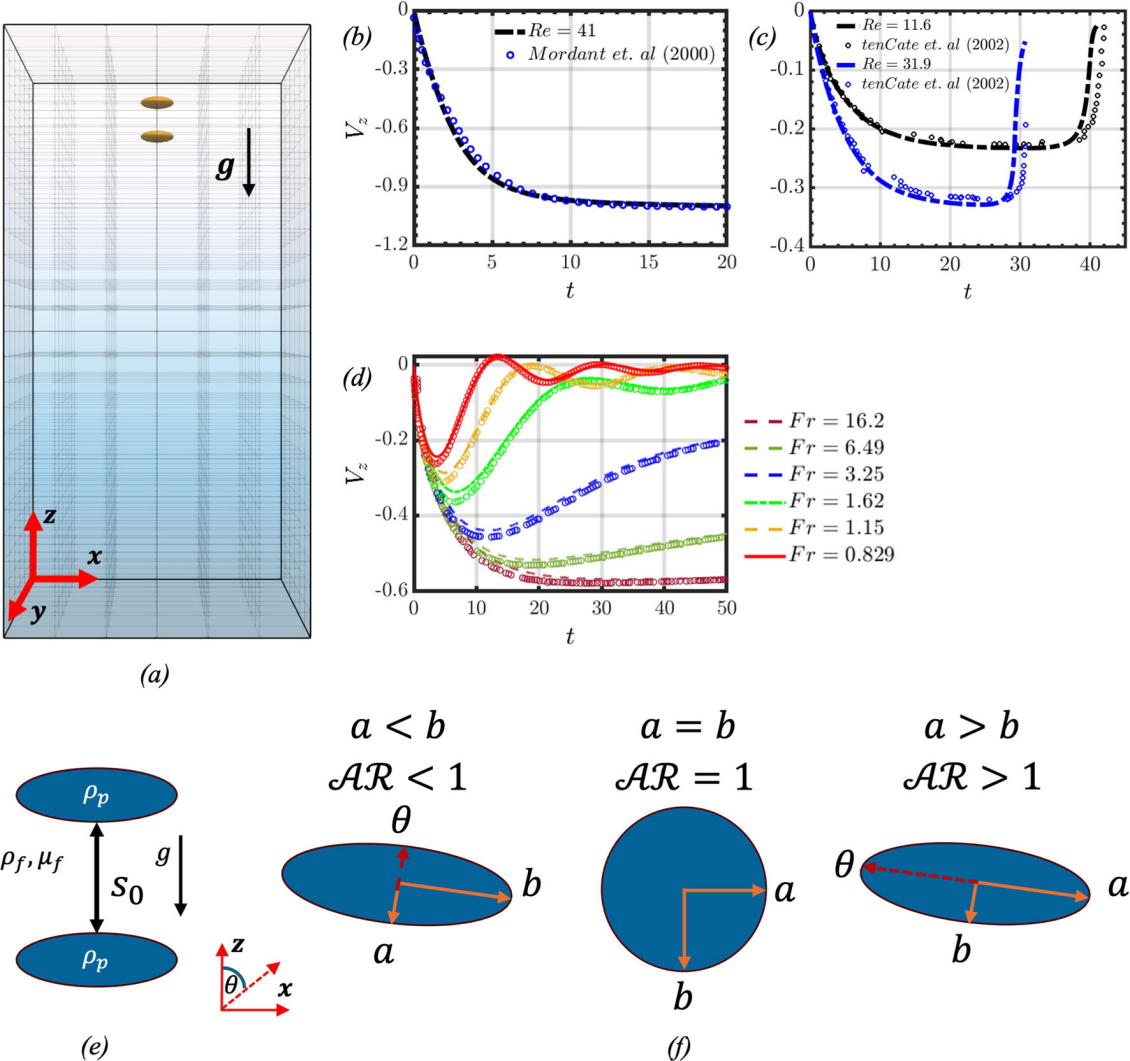
In panel (**a**), the simulation setup for oblate spheroids settling in-line is presented showcasing the subdomain decomposition and the background colour map illustrates the linear background density field. Panels (**b**) and (**c**) compare our numerical predictions with those of [37] and ten Cate et al. [57], respectively, for a sphere settling in a homogeneous fluid. Panel (**d**) shows a similar comparison with the results of [20] for a sphere of diameter $D_p$ settling in a linearly stratified fluid with $Re_s=\rho _0 V_s D_p/\mu _f=14.1$ and different stratification strengths and Froude numbers, *Fr*, where $V_s$ is the Stokes settling velocity. In panel (**e**), a schematic of the initial separation distance between the leading and trailing particles $(s_0)$ is presented. The polar radius of the spheroid is denoted as (*a*), and the equatorial radius as (*b*). In panel (**f**), a schematic of the definitions of the aspect ratio $(\mathcal{A}\mathcal{R}=a/b)$ and the polar angle $(\theta )$ is presented, where $\mathcal{A}\mathcal{R}<1$ is for an oblate spheroid, $\mathcal{A}\mathcal{R}=1$ is for a sphere, and $\mathcal{A}\mathcal{R}>1$ is for a prolate spheroid

The particle shape is characterised by its aspect ratio $\mathcal{A}\mathcal{R}$. The aspect ratio is defined as $\mathcal{A}\mathcal{R}=a/b$ where *a* and *b* denote the particle’s polar and equatorial radii, respectively. An illustration of the axes for the spheroids including a comparison with a sphere can be seen in Fig. 1. For the majority of the discussion, the aspect ratios considered for oblate and prolate spheroids are $\mathcal{A}\mathcal{R}=1/3$ and $\mathcal{A}\mathcal{R}=3$, respectively. The centroid of the trailing particle is initially positioned at two particle diameters below the top of the domain. Unless otherwise stated, the leading particle’s initial position is one particle diameter (from surface to surface) below the trailing particle $(s_0=1)$, which can be seen in Fig. 1. The initial orientation of the spheroids is kept such that the major axis is normal to the direction of gravity. The spheroids always start from rest, and settle in a quiescent background fluid.

The numerical method involves temporally discretising Eq.  (2) into the following form

14 $$\begin{aligned} \begin{aligned} \frac{{\textbf {u}}^{n+1}-{\textbf {u}}^n}{\Delta t}=-({\textbf {u}}\cdot \nabla {\textbf {u}})^n+\frac{1}{\rho ^n}[{\textbf {G}}^n + {\textbf {V}}^n -\nabla p], \end{aligned} \end{aligned}$$

where ${\textbf {G}}$ refers to the gravitational and fluid–structure interaction forces, and ${\textbf {V}}$ refers to the viscous forces, respectively. Here, the fluid–structure interaction force may be computed similarly to the work of [22] as ${\textbf {F}}_{FSI}=\rho \left( \frac{{\textbf {u}}_s -{\textbf {u}}}{\Delta t}\right)$, and it can be considered as directly imposing the particle velocity on the solid boundary, which is equivalent to applying the fluid–structure interaction force inside the solid structure [42]. The momentum solver computes the velocity and pressure variables on a fixed and uniform Eulerian mesh by means of Chorin’s Projection method [11]. For spatial discretisation, the well-known staggered mesh, MAC method, is used [27]. The non-linear term is spatially discretised using a second-order essentially non-oscillatory (ENO) scheme [52, 55], whereas the other terms are spatially discretised using standard second-order centred differences. The time integration of Eq. (14) is split into two sub-steps. An intermediate unprojected velocity, ${\textbf {u}}^*$, is first calculated neglecting the pressure gradient:

15 $$\begin{aligned} \begin{aligned} \frac{{\textbf {u}}^*-{\textbf {u}}^n}{\Delta t}=-({\textbf {u}}\cdot \nabla {\textbf {u}})^n+\frac{{\textbf {G}}^n + {\textbf {V}}^n}{\rho ^n}, \end{aligned} \end{aligned}$$

followed by the calculation of the final velocity, ${\textbf {u}}^{n+1}$,

16 $$\begin{aligned} \begin{aligned} \frac{{\textbf {u}}^{n+1}-{\textbf {u}}^*}{\Delta t}=-\frac{1}{\rho ^n}\nabla p, \end{aligned} \end{aligned}$$

where $\Delta t$ is the time step. By enforcing the divergence-free condition on ${\textbf {u}}^{n+1}$, the elliptic pressure Poisson equation given by,

17 $$\begin{aligned} \nabla \cdot \left( \frac{1}{\rho ^n} \nabla p\right) =\frac{\nabla \cdot {\textbf {u}}^*}{\Delta t}, \end{aligned}$$

is solved using a multigrid iterative method whence ${\textbf {u}}^{n+1}$ is obtained:

18 $$\begin{aligned} \begin{aligned} {\textbf {u}}^{n+1}={\textbf {u}}^*-\frac{\Delta t}{\rho ^n}\nabla p. \end{aligned} \end{aligned}$$

The temporal integration scheme for all the simulations performed is based on a second-order Gear method [59], with implicit solution of the second-order diffusive terms [32]. The time step, $\Delta t$, at each temporal iteration is set to satisfy the following criterion:

19 $$\begin{aligned} \begin{aligned} \Delta t=\text {min}\{\Delta t_{\text {CFL}},\Delta t_{\text {{vis}}}\}, \end{aligned} \end{aligned}$$

where $\Delta t_{\text {CFL}}$, and $\Delta t_{\text {{vis}}}$ are the Courant-Friedrichs-Lewy (CFL) and viscous time-steps, respectively, defined as follows

20 $$\begin{aligned} \begin{aligned}&\Delta t_{\text {CFL}}&\equiv \text {min}_j\left( \text {min}_{\text {domain}}\left( \frac{\Delta x_j}{u_j}\right) \right) ;&\hspace{1em}\Delta t_{\text {{vis}}}&\equiv \text {min}\left( \frac{\rho _p}{\mu _p},\frac{\rho _f}{\mu _f}\right) \frac{\Delta x^2_{\text {min}}}{6}, \end{aligned} \end{aligned}$$

where $\Delta x_{\text {min}}=\text {min}_j(\Delta x_j)$ is the minimum size *x* for cell *j*.

We perform highly resolved numerical simulations of the governing equations in a three-dimensional rectangular Cartesian domain with dimensions $L_x\times L_y \times L_z=12D_p \times 12D_p \times 24D_p$, as shown in panel (a) of Fig. 1. The computational domain is divided into parallel subdomains, which are each uniformly discretised by a $64\times 64 \times 32$ finite-difference mesh. Unless otherwise stated, the mesh size used in this study is $384 \times 384 \times 768$, which corresponds to a resolution of $D_p/\Delta x=32$. This resolution is sufficient to accurately capture the particle settling dynamics and the transient jet dynamics, as confirmed by tests conducted at a lower resolution of $D_p/\Delta x=20$ with less than $3\%$ variation in particle dynamics (not presented herein). Periodic boundary conditions are applied on the side boundaries, and the velocity boundary condition at the top and bottom boundaries correspond to free slip and no-penetration.

The numerical method used in this work has been extensively validated with works found in the literature for a sphere settling in both a homogeneous and stratified fluid, as seen in Fig. 1b–d. Two different experimental works are considered when validating the numerical method for a sphere settling in a homogeneous fluid, and they are the works of [37] and ten Cate et al. [57]. These works consider a range of Reynolds numbers $$(Re=\rho _f V_f D_p/\mu _f)$$ between 11.6 and 41, and the velocity profile of the settling sphere, which is normalised by $$\sqrt{g D_p}$$, matches the experimental results very well. The simulation setup matched those implemented by [5], and the minimum resolution used for these cases was $$D_p/\Delta x=20$$. Furthermore, the numerical method was validated with the computational works of [20] for a settling sphere in a linearly stratified fluid (panel d of Fig. 1) for different *Fr* numbers. The simulation setup matched what was utilised in their work $$(Re_s=\rho _0 V_s D_p /\mu _f=14.1,$$ $$Sc=700)$$, and the settling velocity profiles are in excellent agreement. Note that in the works of [20], the velocity of the sphere is normalised by the Stokes terminal velocity. These validation works inspire confidence in the accuracy and reliability of our computational approach. We also provide in Appendix A a comparison of our predictions with those associated with a Boussinesq fluid to demonstrate the connection between our model, which is valid in the limit of weak diffusion of the stratifying agent and the Boussinesq approximation [20].

**Fig. 2:**
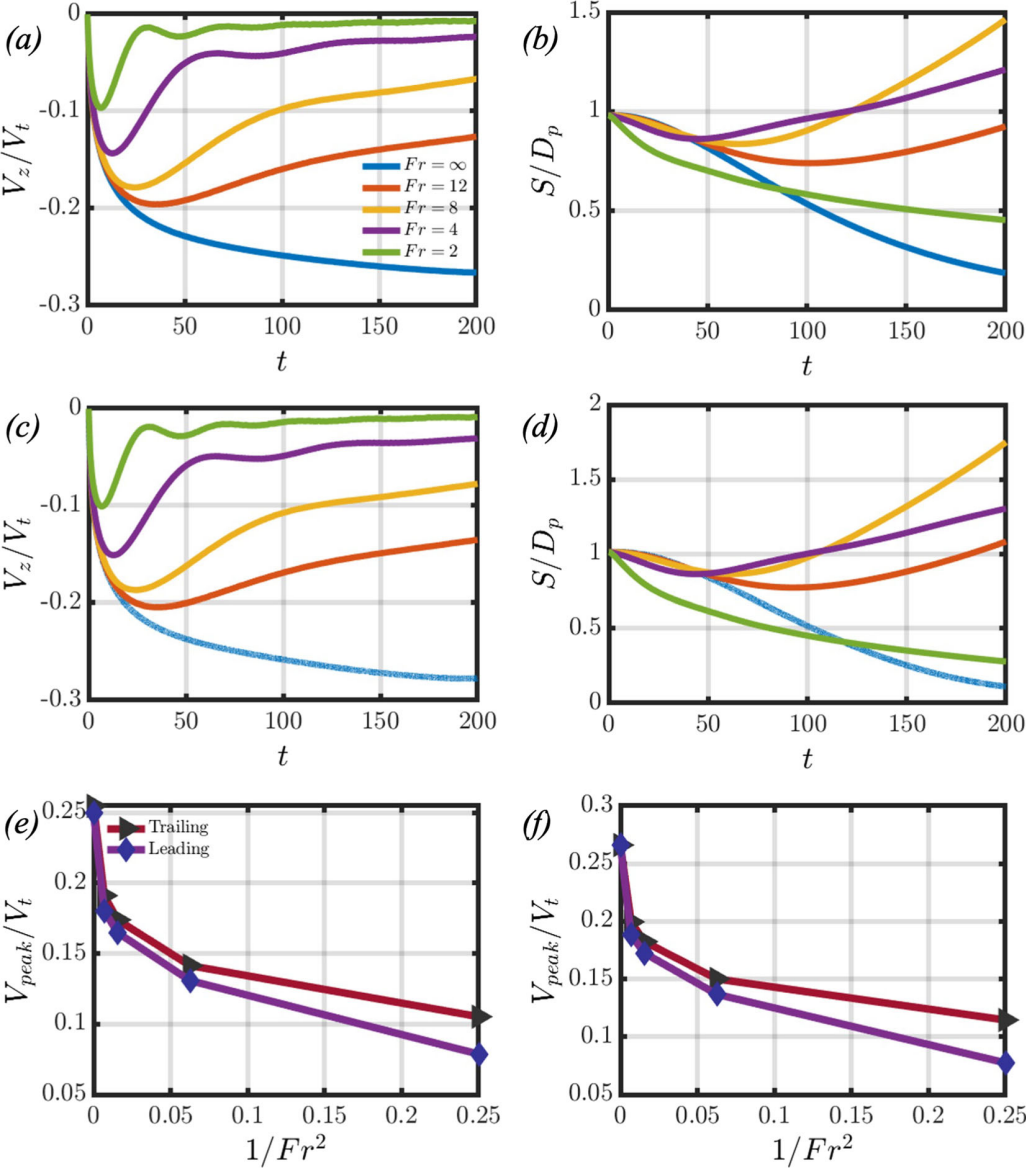
Panels (**a**) and (**b**) present the temporal evolution of the average settling velocity and the normalised distance between the particle surfaces for an oblate particle pair settling in a background fluid of different *Fr*. Panels (**c**) and (**d**) present the same results for a prolate particle pair. Panels (**e**) and (**f**) showcase the difference in peak velocities between the leading and trailing particles across different *Fr* for the oblate and prolate pairs, respectively

## Results and discussion

In this section, we present the computational results of the pairwise interaction between an initially in-line pair of spheroidal particles settling in a linearly stratified fluid. The investigation considers the effect of the *Fr* number on the interaction dynamics, while also considering the particle aspect ratios and the initial separation distance $s_0$ (panel (e) of Fig. 1). The particles are initially oriented ‘broad-side on’, which corresponds to when the major axis is normal to the direction of gravity, such that the polar angle $\theta$ made by the spheroid’s polar axis (*a*) with the *z*-coordinate is initially $0^\circ$ $(90^\circ )$ for an oblate (prolate) spheroid (panel (f) of Fig. 1). We will use the term ‘edge-wise on’ to refer to when the particles’ major axis is aligned with the direction of gravity, such that the polar angle corresponds to $90^\circ$ $(0^\circ )$ for an oblate (prolate) spheroid.

### Settling dynamics

Let us first examine the settling dynamics of the particle pair by considering the effects of the background fluid density gradient when $Ga=4.65$. In Fig. 2, panels (a) and (c) showcase the temporal evolution of the average velocity profiles when varying the *Fr* number for oblate and prolate spheroids, respectively. The time-dependent average velocity profiles are calculated by

21 $$\begin{aligned} V_z=\frac{1}{2}(V_z^{l}+V_z^{t}), \end{aligned}$$

where $V_z^{l}$ and $V_z^{t}$ refer to the settling velocities of the leading and trailing particles, respectively. In the case of a homogeneous fluid $(Fr=\infty )$, the particles start from rest and accelerate until they attain a terminal velocity. The particles then continue to settle at that terminal velocity until reaching the bottom of the domain. However, in the case of a stratified fluid, the particles start from rest at a position where their density is greater than that of the surrounding fluid. The particles accelerate, and as the background fluid density increases, the buoyancy force acting on the spheroids will oppose their settling motion. Therefore, the particles attain a peak velocity, and the balance of drag and buoyancy forces causes the particles to decelerate.

When considering the effect of increasing the stratification strength (decreasing *Fr*), it can be seen in panels (a) and (c) of Fig. 2 that the average peak velocity decreases with *Fr*. This occurs because the effects of buoyancy more efficiently inhibit the settling motion of the spheroidal pair, and aligns with findings from other studies on particles settling in stratified fluids [8, 20, 39, 54]. Figure 2 indicates that for both oblate (panel (e)) and prolate (panel (f)) spheroidal pairs, the gap in peak velocities between the leading and trailing particles grows as the stratification strength increases. Moreover, the trailing particle consistently attains a larger peak velocity. Panels (b) and (d) of Fig. 2 showcase the temporal evolution of the normalised distance $(S/D_p)$ between the leading and trailing particles for oblate and prolate spheroids, respectively. In the homogeneous case, the particles tend to draft as they settle. This is due to the trailing particle being attracted into the wake of the leading particle, thus decreasing the distance between them.

In contrast to the case of larger *Ga* numbers studied by [2], however, the two particles do not come in contact. When introducing stratification effects, it can be seen that the two particles draft initially at moderate stratification, then tend to separate. The drafting duration increases with *Fr* number when considering $Fr\ge 4$. Post the drafting phase, the particles tend to separate such that their separation distance increases at later times when decreasing the *Fr* number. This observation is true for $Fr=12$ and $Fr=8$, but no longer remains valid at $Fr=4$. Furthermore, when $Fr=2$, it is found that the particles tend to draft and do not separate at later times. This reversal is attributed to the increased buoyancy force acting on the particle pair at lower *Fr* numbers, which decreases the settling velocity of the spheroidal pair as previously observed. A deeper exploration of the complexities behind these observations will be presented in Sect. 3.2.

**Fig. 3:**
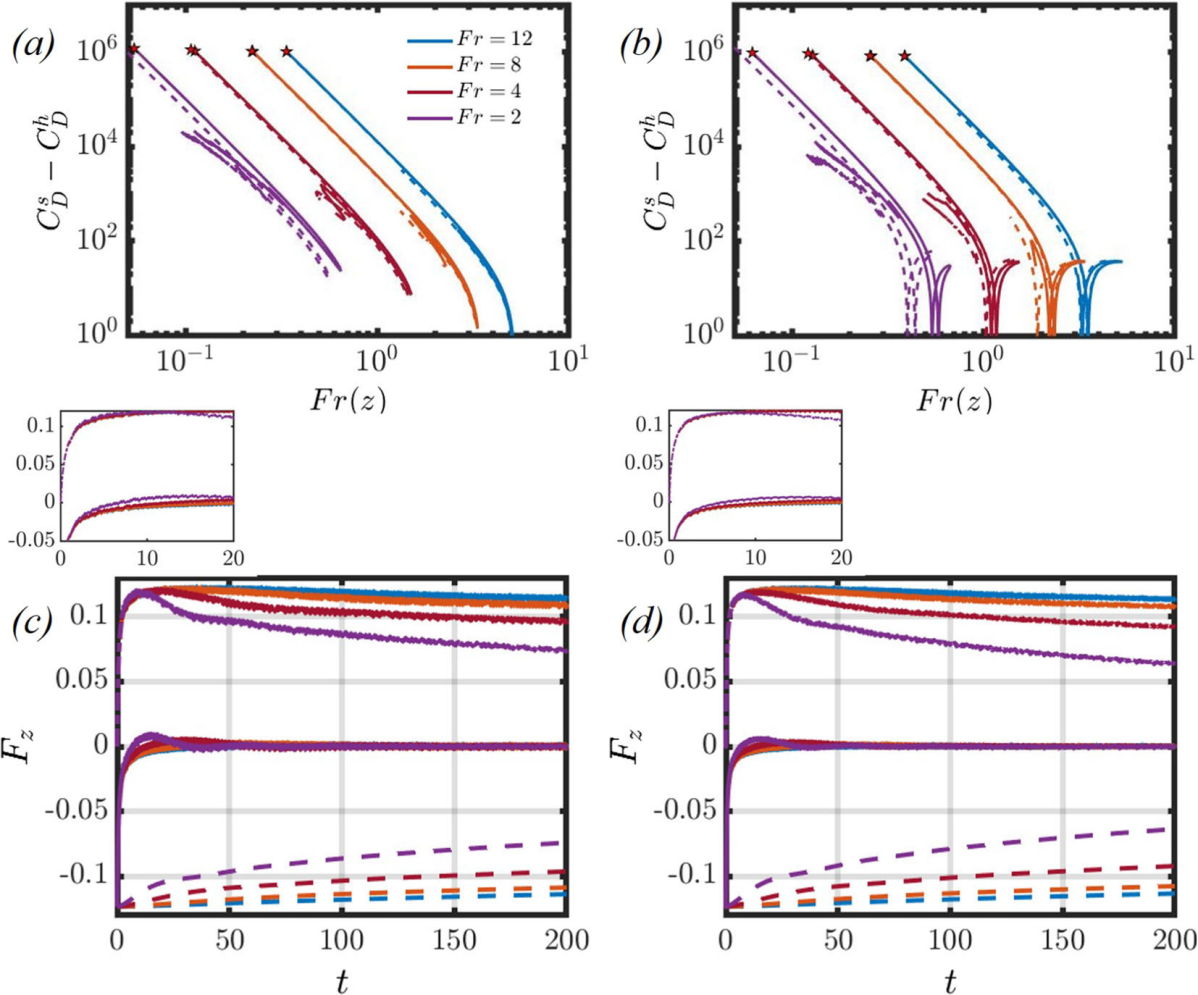
Difference between the stratification and homogeneous drag coefficients for different *Fr* for an oblate and a prolate spheroidal pair, shown in (**a**) and (**b**), respectively. The dashed and solid lines correspond to the leading and trailing particles, respectively. In panels (**c**) and (**d**), the temporal evolution of the buoyancy (dashed), net (solid), and hydrodynamic (dash-dotted) forces are plotted for an oblate and prolate pair, respectively

The literature reveals that stratification significantly increases the drag experienced by a settling particle [20, 36, 39, 60]. If we consider the dynamics of a spheroidal particle settling due to gravity, the equation of motion can be written as

22 $$\begin{aligned} \rho _p \mathcal {V} \frac{\text {d} {\textbf {u}}_p}{\text {d}t}= \rho _p \varvec{g}\mathcal {V} -\int _{\mathcal {V}} \rho _f \varvec{g} d\mathcal {V} +\int _{\mathcal {S}} \varvec{\tau \cdot }  {\textbf {n}} dS+ F_A + F_H, \end{aligned}$$

where $\varvec{\tau }=-p\textbf{I}+\mu (\varvec{\nabla } {\textbf {u}}+\varvec{\nabla }  {\textbf {u}}^T)$ is the stress tensor, $F_A$ is the added mass force, and $F_H$ is the history force. The first and second terms on the right-hand side account for the buoyancy force, and the third term accounts for the hydrodynamic forces. Studies have shown that the added mass and history forces are negligible when compared to the buoyancy and hydrodynamic forces for a particle settling in a stratified fluid [20, 36, 39]. To measure the extent of drag due to stratification, we presume that the spheroid settles in a quasi-steady state and neglect the added mass and history forces. A stratification drag coefficient can be introduced as [60]:

23 $$\begin{aligned} { C_D^s=\frac{4(\rho _p/\rho _f(z) -1)gD_p}{3u_p(z)^2 \mathcal{A}\mathcal{R}^{\alpha }},} \end{aligned}$$

where $\rho _f(z)$ represents the unperturbed background fluid density profile, and $u_p(z)$ denotes the particle velocity at the particle position *z*. The parameter $\alpha$ is taken as $-2/3$ for an oblate spheroid, and 1/3 for a prolate spheroid [2]. Furthermore, several different parameters may also be introduced as a function of the particle’s position,

24 $$\begin{aligned} \Omega (z)=\rho _p/\rho _f(z), \end{aligned}$$

25 $$\begin{aligned} Re(z)=\frac{|u_p(z)|D_p}{\nu }, \end{aligned}$$

where $\Omega (z)$ is the density ratio, $\nu$ is the kinematic viscosity, and *Re*(*z*) is the local Reynolds number. The local Froude number *Fr*(*z*) may be defined as $\sqrt{Re(z)/Ri}$, where $Ri=\gamma g D_p^3 /V_t \mu _f$ is the Richardson number.

[33] provide a drag coefficient for a spheroid settling in a homogeneous fluid $(C_D^h)$, valid for $1 \le Re_p \le 200$ and $0.4 \le \mathcal{A}\mathcal{R} \le 4$ with an error of less than $\pm \, 4\%$:

26 $$\begin{aligned} C_D^h = \frac{24\mathcal{A}\mathcal{R}^{0.49}}{Re(z)}\left[ 1.05+0.152Re(z)^{0.687} \mathcal{A}\mathcal{R}^{0.671}\right] . \end{aligned}$$

Note that when $\mathcal{A}\mathcal{R}=1$, this equation corresponds to a modified form of the drag coefficient formula for spheres in a homogeneous fluid found by [49].

**Fig. 4:**
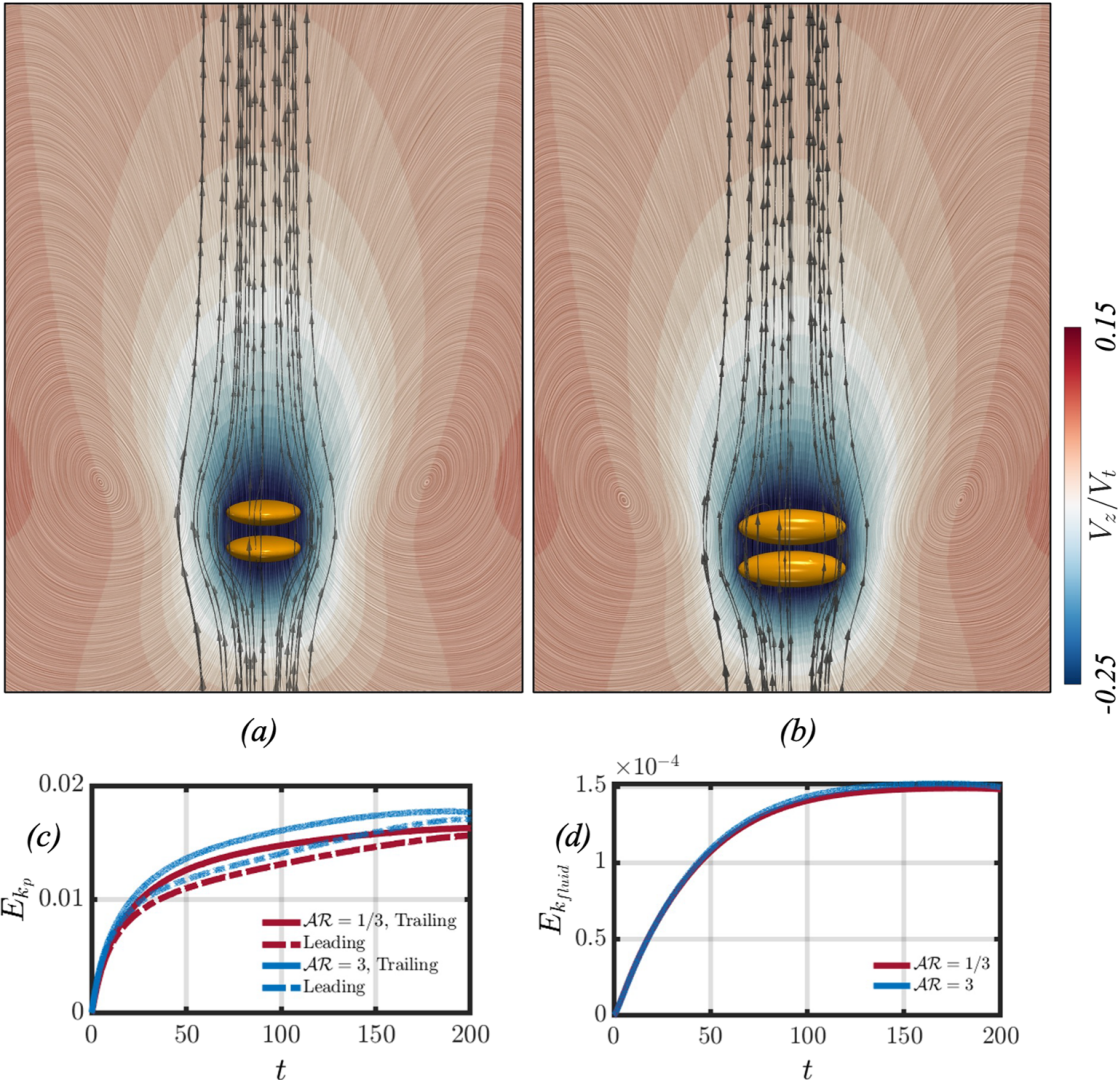
Panels (**a**) and (**b**) showcase the flowfield for a pair of (**a**) oblate and (**b**) prolate particles settling in a homogeneous fluid, where the background colourmap depicts the *z*-velocity field. The background streamlines presented are in the stationary reference frame, and the streamlines are in the reference frame of the leading particle. Panels (**c**) and (**d**) showcase the time history of the particle and fluid kinetic energies, respectively. The particle and fluid kinetic energies are normalised by their respective masses $(MV_t^2)$

In panels (a) and (b) of Fig. 3, the difference between the stratified and homogeneous drag coefficients is measured $(C_D^s - C_D^h)$ when varying *Fr* for an oblate and prolate spheroidal pair, respectively. As seen by [39], the particles initially start from rest and accelerate, leading to an increase in *Fr*(*z*). During the acceleration phase, the disparity between the stratified drag coefficient, $C_D^s$, and the homogeneous drag coefficient, $C_D^h$, diminishes, reaching its lowest point as the particles attain their maximum velocity. As the influence of buoyancy intensifies, the drag induced by stratification surges, causing the particles to slow down. The results show that the stratification drag coefficient is orders of magnitude larger than its homogeneous counterpart. The difference between the two drag coefficients reveals the importance of accounting for the additional drag due to stratification when studying particulate transport in fluids with density gradients.

In panels (c) and (d) of Fig. 3, the buoyancy (dashed lines), hydrodynamic (dashed-dotted lines), and net force (solid lines) acting on the trailing particle are measured for different *Fr* for the oblate and prolate cases, respectively. The buoyancy and hydrodynamic forces are calculated using equation 22, and the net force is taken as the sum of the two contributions. In a homogeneous fluid, the buoyancy force remains constant and can be determined by $F_B=(\rho _p-\rho _0)gV$. As the particle accelerates over time and reaches its peak velocity, a balance is established between the hydrodynamic forces and the buoyancy force, and the particle settles at this constant terminal velocity.

In contrast, within a stratified fluid, the initial magnitude of the buoyancy force is greatest since the disparity in density between the particle and the surrounding fluid is at its maximum. This creates a negative net force at the outset, prompting the particle to accelerate. As the particle accelerates, hydrodynamic forces build up and the magnitude of the buoyancy force diminishes. The peak velocity of the particle is achieved when the hydrodynamic force exceeds the buoyancy force. At this point, the buoyancy force is insufficient to offset the hydrodynamic force, leading to a deceleration of the particle when the net force becomes positive. If the particle reaches a position where it is neutrally buoyant, its settling motion will stop, as there is no longer any net force acting on the particle.

### Flow field

This section will build upon the findings of Sect. 3.1 and investigate the details of the flow field, particle orientation, and interaction between the particles when $Ga=4.65$.

**Fig. 5:**
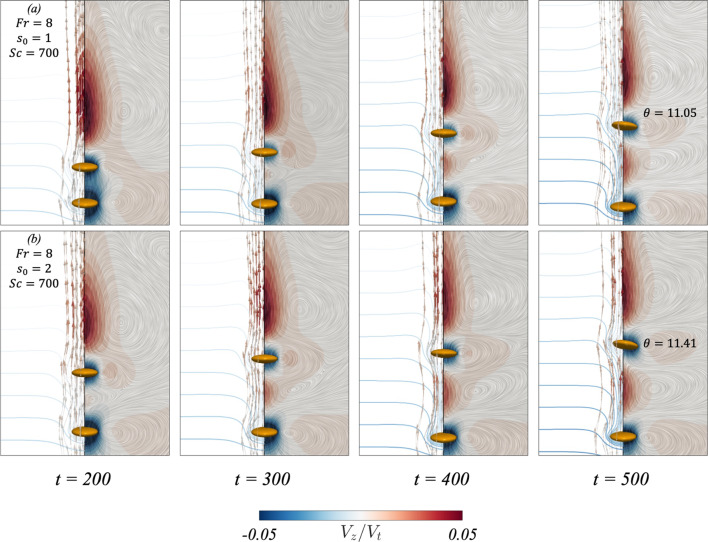
Results for the dynamic interaction between two settling oblate spheroids under different conditions. From left to right of every row, each panel showcases the flow field (right side) and streamlines (left side) in the reference frame of the leading particle. The left side of each panel also presents contours depicting the distortion of the pycnoclines, which are coloured such that the dark blue represents a heavier fluid. The difference in density between two adjacent pycnoclines is $\Delta \rho _f /\gamma D_p =1.14$. The colour map in the flow field illustrates the fluid velocity. Rows (**a**–**b**) correspond to $(Ga, \mathcal{A}\mathcal{R}, Fr, s_0)=(4, 1/3, 8, 1), (4, 1/3, 8, 2)$, respectively

**Fig. 6:**
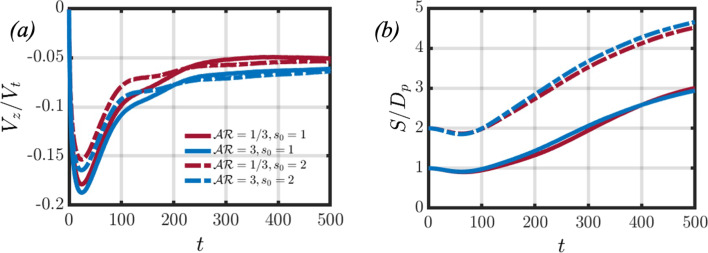
This figure showcases the settling dynamics (**a**–**b**) when varying $s_0$ for both an oblate and prolate particle pair. The results showcase the normalised settling velocities and separation distances between the two spheroids

Let us first consider the interaction between spheroids settling in a homogeneous fluid. Panels (a) and (b) of Fig. 4 showcase the flow fields for an oblate and prolate spheroidal pair settling in the absence of stratification, respectively. The analysis of the flow fields indicates that the settling behaviour of the spheroidal particle pair is axisymmetric, featuring areas of negative velocity in the fluid surrounding the particles. In panels (c) and (d), the kinetic energy for both the particle pair and the surrounding fluid is depicted. The total kinetic energy $E_k$ of the system is defined as follows,

27 $$\begin{aligned} E_k=\frac{1}{2}\rho \int _V (u^2+v^2+w^2) dV, \end{aligned}$$

and may be separated into two different contributions. The two contributions are the kinetic energy of the particles and the background fluid, such that $E_k=E_{k_p}+E_{k_{fluid}}$. It is observed that the kinetic energy associated with the trailing particle is greater than that of the leading particle at later times. This difference is attributed to the pressure gradient induced by the leading particle’s wake, which propels the trailing particle to higher settling velocities. As for the fluid’s kinetic energy, it increases until reaching a steady state, signifying that the particle pair has reached a constant terminal velocity.

**Fig. 7:**
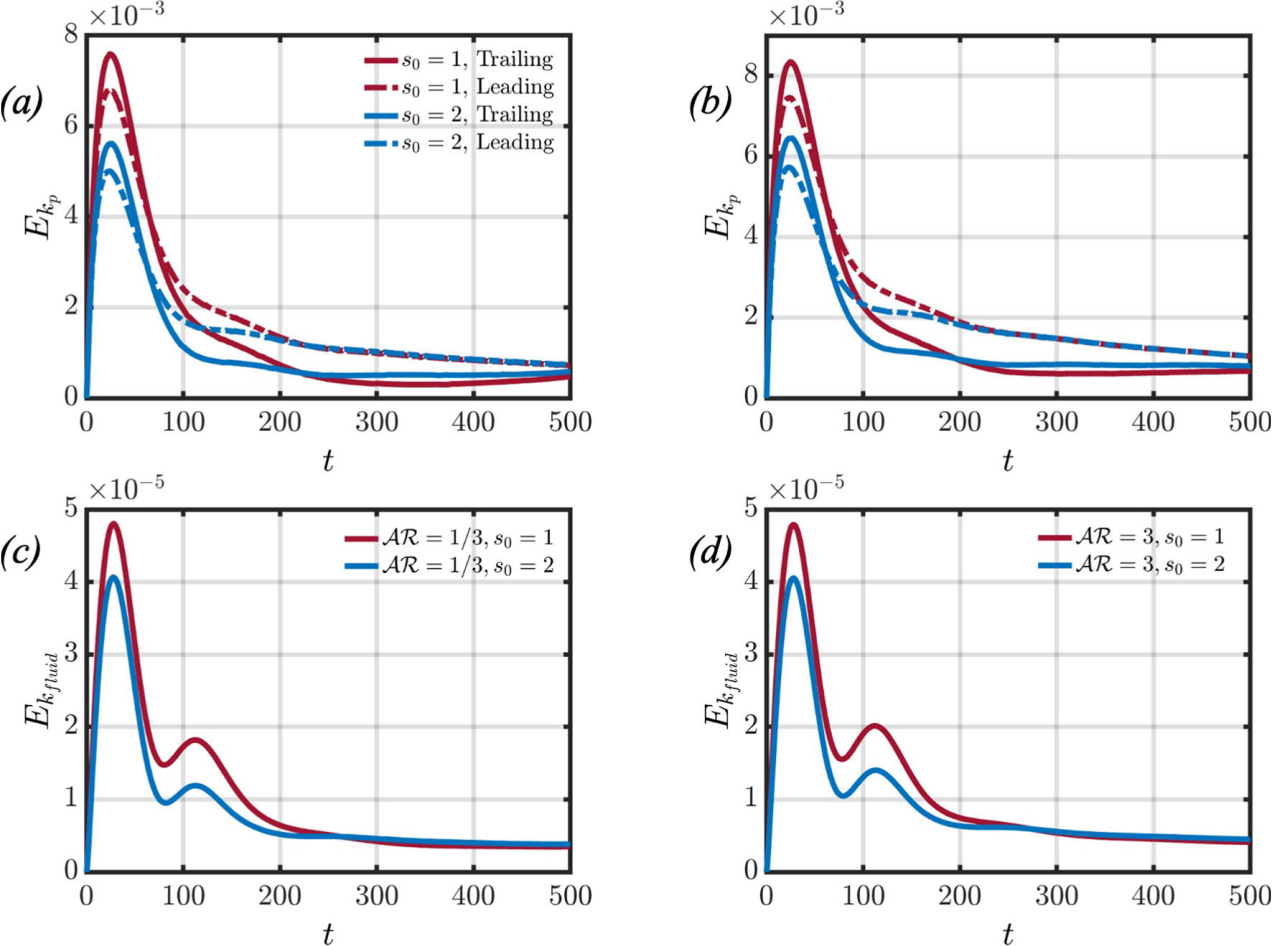
This figure showcases the particle and fluid kinetic energies when varying the initial separation distance for an oblate (panels (**a**) and (**c**)) and prolate (panels (**b**) and (**d**)) particle pair

When considering the flow field for an oblate spheroidal pair settling in a stratified fluid, Fig. 5 showcases the flow field at different times for two cases. These two cases correspond to a base case with $(Fr, s_0, Sc)=(8, 1, 700)$ in row (a), and increasing the initial separation distance in row (b). When the spheroids settle in a stratified fluid, they drag along with them lighter fluid which affects their settling velocities as seen in Sect. 3.1. The dragged fluid can be visualised by the distortion of the pycnoclines on the left-hand side of each panel. This fluid moves along the trailing spheroid until it reaches its rear stagnation point, where it forms an upward jet that returns this light fluid to its neutrally buoyant position [38]. The upward jet can be identified as the region with positive velocity (coloured in red) in Fig.  5. At $t=200$, it can be seen in row (a) that the fluid neighbouring the particle has a negative velocity. In time, the two particles separate due to viscous dissipation more efficiently limiting the inertial effects at this *Ga* number, such that the particles do not come in contact [17]. As the particles separate, the fluid region of negative velocity between the leading and trailing particles diminishes as seen at $t=300$. The dragged lighter fluid by the leading particle then forms an upward jet in the region between the particle pair, further increasing the separation distance between the leading and trailing particles as seen in panel (b) of Fig. 6. This causes a sharp decrease in the trailing particle’s settling velocity, as seen in panel (a) of Figs. 6 and 7. [39] and [36] have found that an oblate spheroid settling in a stratified fluid reorients to its edgewise orientation when the particle’s velocity decreases below some threshold value. In row (a) of Fig. 5, at the last time step, it can be seen that the trailing particle begins to reorient to its edgewise orientation, where $\theta \approx 11^\circ$.

**Fig. 8:**
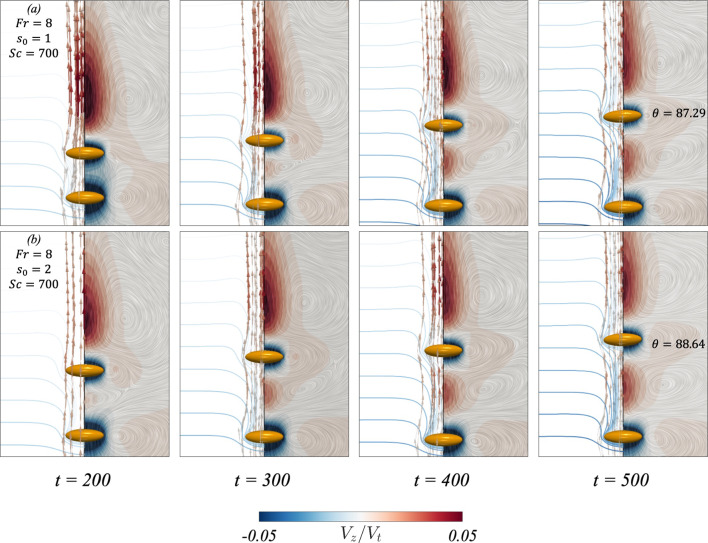
Results for the dynamic interaction between two settling prolate spheroids under different conditions. From left to right of every row, each panel showcases the flow field (right side) and streamlines (left side) in the reference frame of the leading particle. The left side of each panel also presents contours depicting the distortion of the pycnoclines, which are coloured such that the dark blue showcases a heavier fluid. The difference in density between two adjacent pycnoclines is $\Delta \rho _f /\gamma D_p =1.14$. The colour map in the flow field illustrates the fluid velocity. Rows (**a**–**b**) correspond to $(Ga, \mathcal{A}\mathcal{R}, Fr, s_0)=(4, 1/3, 8, 1), (4, 1/3, 8, 2)$, respectively

**Fig. 9:**
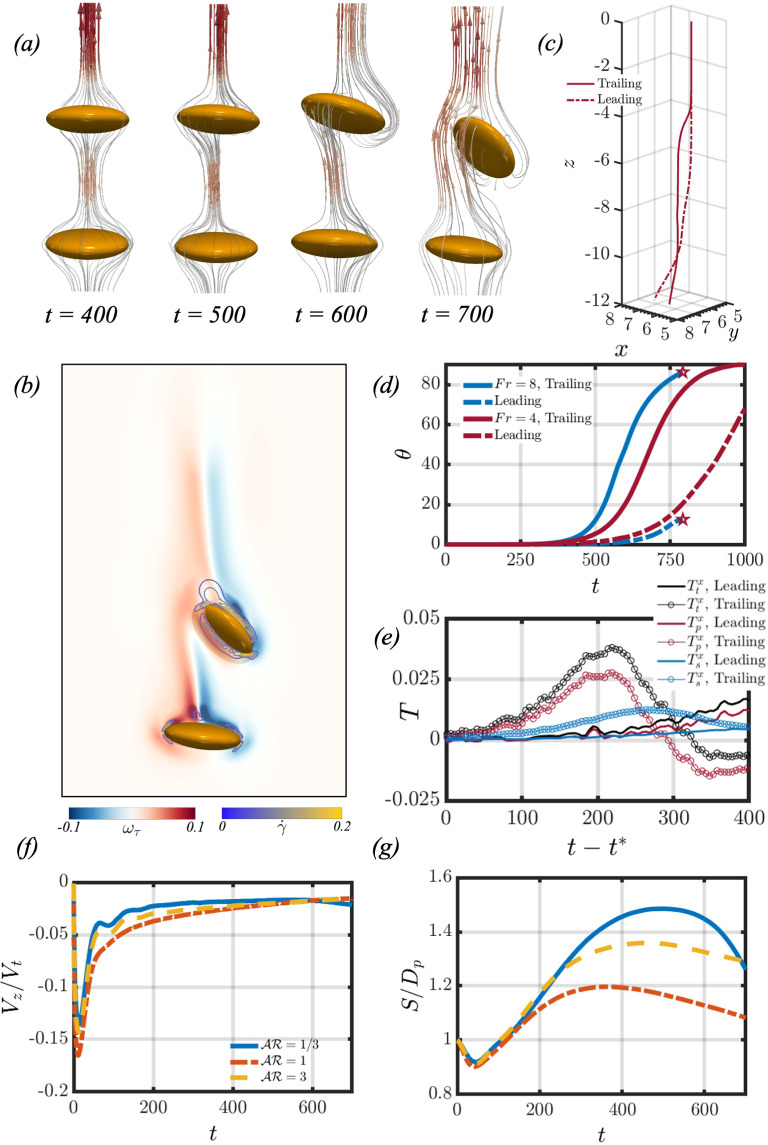
Panel (**a**) of this figure shows the evolution of the asymmetric side escape of the trailing particle for an oblate spheroidal pair when $Fr=4$. At $t=700,$ panel (**b**) showcases the baroclinic vorticity contribution and contours of the shear rate near the particle surface. In panel (**c**), the three-dimensional trajectories of the trailing and leading particles are depicted, and panel (**d**) showcases the temporal evolution of the polar angle $\theta$ for two different *Fr* numbers for an oblate spheroidal pair. Note that the pentagram in panel (**d**) is for the simulation end time. Panel (**e**) shows the evolution of the torques acting on the leading and trailing particles when $Fr=4$. Panels (**f**) and (**g**) showcase the settling dynamics when $Fr=4$ and varying the aspect ratio

In panel (b) of Fig. 5, the effect of increasing the initial separation distance between the two particles is considered. At the initial time step, the leading particle is surrounded by fluid that is heavier when compared to the base case. As a result, the leading particle is subjected to a larger buoyancy force, which limits its attainable peak velocity, as seen in panel (a) of Fig.  6. The decrease in peak velocity leads to a subsequent decrease in peak particle and fluid kinetic energies, as found in panels (a) and (c) of Fig. 7. However, a similar flow structure is found when compared to the base case; however at $t=200$, the region of negative fluid velocity between the particle pair is no longer present. This can be explained by the wake of the leading particle causing a smaller pressure difference between the front and back of the trailing particle, such that the trailing particle’s enhanced acceleration is not as significant when compared to the base case. This can be seen by the slightly decreased drafting time between the two cases (panel (b) of Fig. 6) and narrows the gap between the peak kinetic energies of the leading and trailing particles, as seen in panel (a) of Fig. 7. The trailing particle’s kinetic energy then decreases, which increases the separation distance between the particle pair. Therefore, the onset of the upward jet in the region between the particle pair can be found at earlier times $(t=300)$. As the trailing particle’s velocity decreases, it starts to reorient to its edgewise orientation, as seen in the base case.

When considering a prolate particle pair settling in a stratified fluid, Fig. 8 considers the same study of varying the initial separation distance on the pairwise interaction. The overall flow dynamics are found to be similar to the oblate counterpart. There are a few subtle differences, however, that are worth mentioning. Firstly, the upward jet that forms behind the trailing particle, Fig. 8 shows that the jet radius is slightly larger when compared to the previous case. The particle settling velocities, found in panel (a) of Fig. 6 are slightly larger, leading to an increase in the particle kinetic energies (panel (b) of Fig. 7). The increase in particle kinetic energy compared to the oblate counterpart was also found for the homogeneous case, as seen in panel (c) of Fig. 4. Lastly, the reorientation of the trailing particle is not as significant when considering a prolate particle pair, as the polar angle varies by about $2^\circ - 3^\circ$ at $t=500$, compared to $\theta \approx 11^\circ$ for the oblate case.

In Sect. 3.1, we have mentioned that in time, the separation distance between the two particles tends to increase when decreasing *Fr*. Here, we will examine the effect of decreasing the *Fr* number on the settling behaviour. In panel (a) of Fig. 9, the temporal evolution of the streamlines in the reference frame of the leading particle is presented for an oblate spheroidal pair when $Fr=4$. At $t=400$, the separation distance between the two particles is large, as seen in panel (g) of Fig. 9. However, axial symmetry breaks, and the trailing particle tilts clockwise as seen at $t=500$. At this point, the trailing particle stands in the wake of the leading particle with its major axis tilted, which distorts the flow in the gap region between them. The flow grows increasingly asymmetric in this region at $t=600$, and this asymmetry induces a slight clockwise tilt on the leading particle. The trailing particle then drifts laterally, starting to escape the wake of the leading particle. The separation distance between the particles then decreases, as the trailing particle attains its edgewise orientation. This phenomenon is reminiscent of the asymmetric side escape regime found for two in-line rising bubbles studied by [61]. The three-dimensional trajectory of the leading and trailing particles can be appreciated in panel (c), and panel (d) showcases the time history of the polar angle $\theta$ for an oblate spheroidal pair settling when $Fr=4$ and $Fr=8$. Note that when $Fr=8$, the simulation was stopped when the leading particle reached the bottom boundary, which corresponds to $t \approx 750$.

**Fig. 10:**
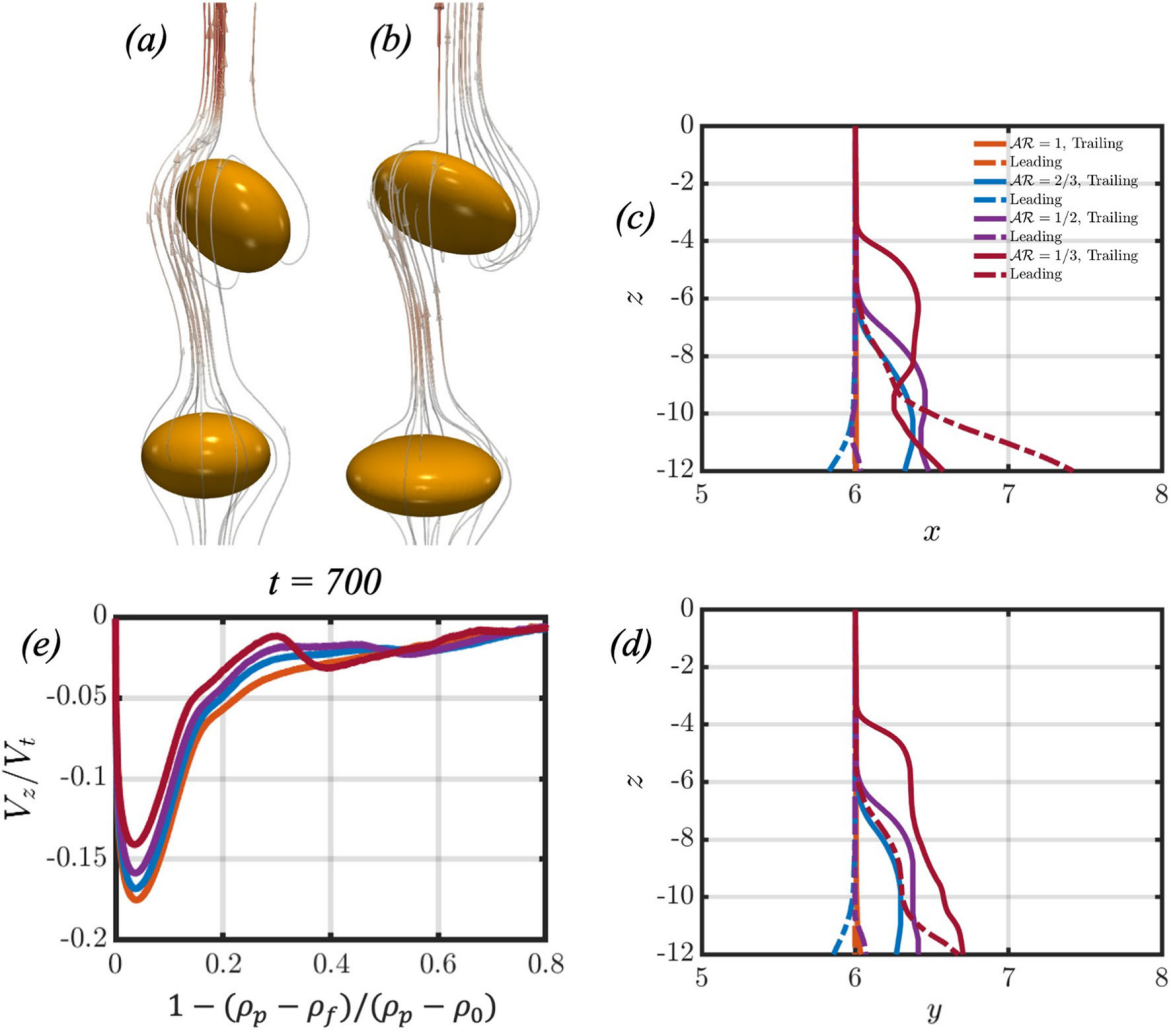
Panels (**a**) and (**b**) showcase the asymmetric side escape of the trailing particle when $Fr=4$ for two different aspect ratios of oblate spheroids. The aspect ratios are $\mathcal{A}\mathcal{R}=2/3$ and $\mathcal{A}\mathcal{R}=1/2$, respectively. Panels (**c**) and (**d**) showcase the side views of the settling trajectories of the particle pair when varying $\mathcal{A}\mathcal{R}$ and $Fr=4$. Panel (**e**) showcases the evolution of the trailing particle’s velocity profile when varying $\mathcal{A}\mathcal{R}$ against the normalised density

The distortion of the isopycnals as the particles settle gives rise to baroclinic vorticity generation $(\frac{1}{\rho _f^2}\nabla \rho _f \times \nabla p)$ due to the misalignment of the density gradient with the direction of gravity. At $t=700$, the baroclinic vorticity is depicted in panel (b) of Fig. 9. Closer to the particle surface, the formation of these vortices induces a high shear rate $\dot{\gamma }=\sqrt{2\varvec{D: D}}$, where $\varvec{D}$ is the rate of strain tensor [18]. Furthermore, the total torque experienced by the spheroids as they settle is composed of two distinct components: the contributions from pressure forces and viscous stresses:

28 $$\begin{aligned} T_p=-\int _{\mathcal {S}}(\varvec{r}-\varvec{r_c})\times (p\varvec{n})dS, \end{aligned}$$

29 $$\begin{aligned} T_s=\int _{\mathcal {S}}(\varvec{r}-\varvec{r_c})\times \mu (\varvec{\nabla }  {\textbf {u}}+\varvec{\nabla }  {\textbf {u}}^T)\cdot {\textbf {n}} dS, \end{aligned}$$

where $\varvec{r}-\varvec{r_c}$ indicates the distance from the particle surface to its centroid. The total torque can then be written as the sum of these two components, such that $T_t=T_p+T_s$. The temporal evolution of the different torque contributions when $Fr=4$ can be found in panel (e), where $t^* \approx 400$ denotes the start of the trailing particle reorientation process. Concentrating on the $x-$component of the torque, the initial reorientation of the trailing particle is primarily driven by torque resulting from pressure forces. Yet, as the trailing particle narrows the gap distance and attains its edgewise orientation, baroclinic vortices intensify, which amplifies the shear stress across its surface. The increase in the shear stress along the particle’s surface gives rise to $T_s$, which opposes the decreasing pressure-induced torque contribution. When the particle slows down and reaches a stable edgewise orientation, the contribution from viscous torque decreases, leading to a reduction in particle rotation. A similar analysis can be made for the leading particle.

In Fig. 8, we can see that the induced reorientation of the trailing prolate particle is not as significant as the oblate counterpart, however, the trailing particle is found to tilt in the anticlockwise direction instead. When decreasing the *Fr* number, panels (f) and (g) of Fig. 9 provide a comparison of the average settling velocities and separation distances for three different aspect ratios. These aspect ratios correspond to the oblate spheroidal pair, prolate spheroidal pair, and a pair of spheres $(\mathcal{A}\mathcal{R}=1)$. When considering $\mathcal{A}\mathcal{R}=3$ and $\mathcal{A}\mathcal{R}=1$, the decrease in distance between the leading and trailing particles when $t \ge 400$ is not as significant as the oblate counterpart. This can be explained by the reduced reorientation found earlier for the prolate particle pair, such that the decrease in separation distance is primarily due to buoyancy leading to a decrease in the leading particle’s settling velocity.

Figure 10 investigates the effect of gradually decreasing the aspect ratio on the trailing particle’s escape when $Fr=4$. Two additional aspect ratios are considered, and they are $\mathcal{A}\mathcal{R}=2/3$ and $\mathcal{A}\mathcal{R}=1/2$. The streamlines at $t=700$ for these two cases can be found in panels (a) and (b) respectively. In both cases, the trailing particle is found to rotate clockwise, inducing an asymmetric flow pattern in the gap region between the two particles. When considering the settling trajectories, panels (c) and (d) provide a side view of the particle pair when varying $\mathcal{A}\mathcal{R}$. For $\mathcal{A}\mathcal{R}=1$, the particles are seen to settle in-line without colliding, as found at low *Ga* numbers by [17]. As the $\mathcal{A}\mathcal{R}$ number decreases, two main observations are made. When decreasing $\mathcal{A}\mathcal{R}$, the magnitude of lateral drift of the trailing particle increases and occurs at a higher vertical position. This can be seen by investigating the side view of the particle trajectories. When considering the settling velocities of the trailing particles, panel (e) shows the evolution of the velocity profile as a function of the normalised density, where the magnitude of the peak velocity was found to increase with increasing $\mathcal{A}\mathcal{R}$.

**Fig. 11:**
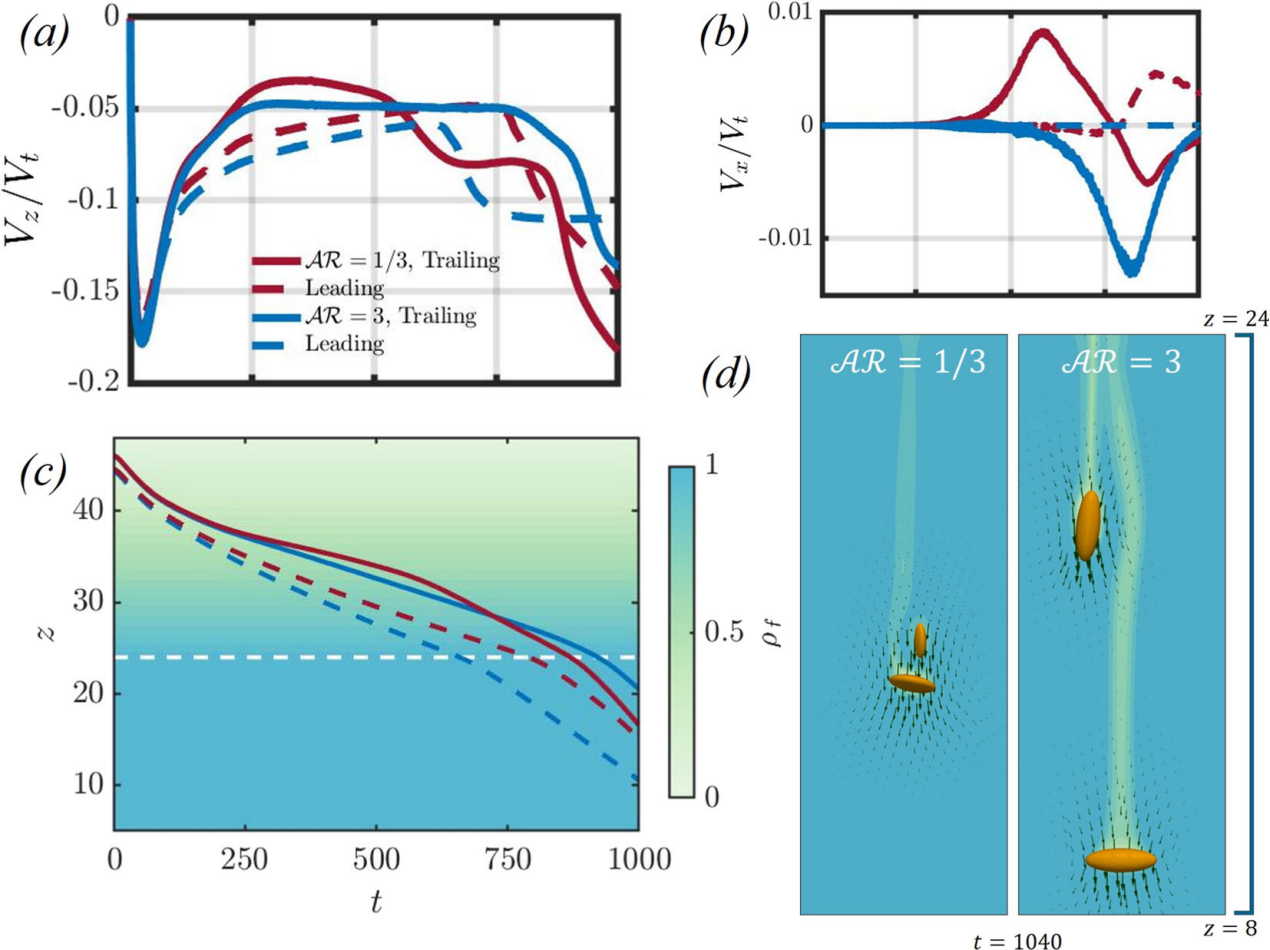
This figure showcases the transient dynamics of an oblate and prolate spheroidal pair settling in a linearly stratified fluid with $Fr=8$, $Sc=700$, and $Ga=4.65$ for a vertical distance of $z=24$, and settling in a fluid with constant density after that for a distance of $z=24$. Panels (**a**) and (**b**) present the *z* and *x* particle velocities, and panel (**c**) showcases the temporal evolution of the vertical position of the spheroidal pair. The dashed white line in panel (**c**) is at $z=24D_p$, which is at the transition from linearly stratified above the line to constant density below the line. In panel (**d**), qualitative results are presented for the two cases after the particles cross the linearly stratified region at $t=1040$

While in a linearly stratified fluid, particles settling due to gravity do not achieve a statistically steady state because of the continuously increasing background fluid density, an asymmetric side escape of the trailing particle at moderate stratification strengths is presented in this work. Previous studies have shown that the reorientation of discs settling in stratified fluids diminishes as they pass through the stratification interface [40]. Conversely, for prolate spheroids, [18] found that as a particle crosses a sharp density interface, it experiences enhanced reorientation to a broad-side on orientation, which diminishes once the particle settles into the denser fluid. Given that the reorientation of the particles past the density interface remains an open question for the specific configuration studied in this work, we include two additional cases to examine the effects of an extended domain on the reorientation and repositioning of these particles. Specifically, we will consider a linearly stratified fluid over a vertical height of $24D_p$, followed by a region of constant fluid density extending another $24D_p$ below the stratified layer. In Fig. 11, we present the (a) settling and (b) horizontal velocities of the particles as they traverse the linearly stratified region. As the particles exit the stratified zone, their settling velocities are expected to increase in magnitude as the lighter fluid column, drawn by the particles, returns to its neutrally buoyant state. Panel (c) illustrates the temporal evolution of the vertical position of the spheroidal particle pair, while panel (d) provides qualitative results for the particle pair at $t=1040$.

For an oblate particle pair, the trailing spheroid attains its edgewise orientation and continues to settle in that orientation beyond the density interface, with a larger settling velocity than the leading particle. The trailing particle also drifts closer to the leading particle, as indicated by the variation in $V_x$ in panel (b). The leading particle maintains an orientation between broad-side on and edgewise on $(\theta \approx 17.9^\circ )$ during its descent past the stratified region. In contrast, for the prolate spheroidal pair, the leading particle continues to settle past the density interface in a broad-side on orientation, while the trailing particle attains an edgewise on orientation. The separation distance between the particles is significantly larger compared to the oblate pair, which can be attributed to the increased settling velocity of the leading prolate particle due to its broad-side on orientation. It is important to note that these findings pertain to a particle pair settling in a stratified fluid with $Sc=700$, corresponding to a salinity-stratified fluid, with particles having an equivalent spherical diameter of 250 $\mu$m. Future work will explore the effects of the diffusivity of the stratifying agent and particle size on their settling behaviour.

## Concluding remarks

This work studies the interaction dynamics for a spheroidal particle pair settling in-line in a linearly stratified fluid. Firstly, the study considers the effects of the stratification strength, characterised by the Froude number (*Fr*), on the settling dynamics when the Galilei number $Ga=4.65$. The particle shape is governed by the aspect ratio $\mathcal{A}\mathcal{R}=1/3$ and $\mathcal{A}\mathcal{R}=3$ for an oblate or prolate spheroid, respectively. The results showcase an initial drafting phase of the trailing particle into the leading particle’s wake, followed by a separation phase of the two particles due to the effects of the stratification. During the drafting phase, a buoyant jet forms behind the trailing particle due to the restoration of the pycnoclines to their neutrally buoyant position. As the particles separate, a secondary jet forms in the region between the leading and the trailing particles, which causes the trailing particle to reorient. The reorientation was found to be more significant for the oblate cases when compared to the prolate cases. For the oblate cases, the secondary jet causes the trailing particle to tilt and escape the wake of the leading particle, leading to an asymmetric side escape regime. Increasing the initial separation distance between the two particles led to an earlier onset of the secondary jet between the two particles. Additional aspect ratios were considered for the oblate cases, such that $\mathcal{A}\mathcal{R}=2/3$ and $\mathcal{A}\mathcal{R}=1/2$ at large *Sc* numbers. At these aspect ratios, the trailing particle was still found to escape the wake of the leading particle, with decreased lateral drift with increasing $\mathcal{A}\mathcal{R}$.

This work mainly focused on moderate stratification strengths $(Fr=4$ to 8) and particle sizes $(D_p=250$ $\mu \text {m})$. These parameters correspond to the settling behaviours of particles as found in the ocean [34]. The ultimate goal of this work is to gain insight into the interaction dynamics between particles as found in geophysical applications and the associated induced mixing, and future directions will include the study of the collective transport of a large number of non-spherical particles in such stratified environments. In this work, the particle pair for each case always had the same initial orientation and the same size and shape, whereas varying these conditions will undoubtedly lead to different interaction dynamics. Ongoing work is considering the collective transport of spherical and non-spherical particles in a stratified ambient fluid.

## Figures and Tables

**Fig. 12:**
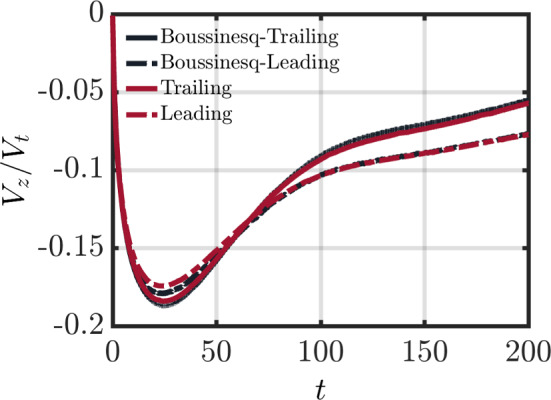
This figure showcases the comparison of the model presented in Sect. 2 with the Boussinesq incompressible Navier Stokes equations

## Appendix A Comparison with the Boussinesq approximation

In this section, we will compare the results obtained with the model presented in Sect. 2 with the Boussinesq approximation. The Boussinesq approximation assumes that the density perturbation is much smaller than the reference density $(|\rho (z)- \rho _0|\ll \rho _0)$. In this case, the momentum and transport equations in the fluid may be rewritten as follows:

A1 $$\begin{aligned}&\displaystyle \rho _0\left( \frac{\partial {\textbf {u}}}{\partial t}+{\textbf {u}}\cdot \nabla {\textbf {u}}\right) =-\nabla {p}+\rho _f{\textbf {g}}+\nabla \cdot \mu \left( \nabla {\textbf {u}}+\nabla {\textbf {u}}^T\right) , \end{aligned}$$

A2 $$\begin{aligned}&\displaystyle \frac{\partial T}{\partial t}+{\textbf {u}}\cdot \nabla T = \nabla \cdot (\kappa _t \nabla T ), \end{aligned}$$

where *T* is the background fluid temperature, and $\kappa _t$ is the thermal diffusivity of the ambient fluid. The Prandtl number, $Pr=c_p \mu _f /\kappa _c$, where $\kappa _c$ is the thermal conductivity and $c_p$ is the specific heat capacity, is used in this case to govern the thermal diffusivity as $\kappa _t=\kappa _c/\rho _0 c_p$. The fluid density then may be related to the background temperature field, such that $\rho _f=\rho _0(1-\beta _f(T-T_0))$, where $\beta _f$ is the thermal expansion coefficient of the ambient fluid.

The comparison between the two results presented in Fig. 12 was for an oblate spheroidal pair with $\mathcal{A}\mathcal{R}=1/3$ where $Fr=8$, $Ga=4.65$, and $Pr=Sc=700$. Note that for a thermally stratified fluid, the no-flux boundary condition on the particle surface is an idealised assumption that the particle’s conductivity is negligible when compared to that of the background fluid [20, 39]. The results show that the velocity profile of the leading and trailing particles closely match the results for the Boussinesq case, which allows our assumption of enforcing incompressibility in the limit of minimal diffusion.
